# O Ecocardiograma Intracardíaco Deve Ser Utilizado em Todo Paciente Submetido a Ablação de FA?

**DOI:** 10.36660/abc.20230290

**Published:** 2023-06-02

**Authors:** Cristiano F. Pisani, Mauricio Scanavacca

**Affiliations:** 1 InCor HC FMUSP São Paulo SP Brasil Unidade Clínica de Arritmia do Instituto do Coração (InCor) do Hospital das Clínicas da FM USP (HC-FMUSP), São Paulo, SP – Brasil

**Keywords:** Arritmias Cardíaca, Fibrilação Atrial, Ecocardiografia/métodos, Ecocardiografia/tendências, Ablação por Cateter, Ecocardiografia Transesofagiana/métodos, Técnicas de Ablação/tendências

A ablação da fibrilação atrial tornou-se uma técnica amplamente utilizada no tratamento dos pacientes com fibrilação atrial nos últimos 20 anos devido ao melhor conhecimento dos seu mecanismos fisiopatológicos, evolução nas técnicas de isolamento das veias pulmonares e redução das complicações associadas ao procedimento.^1^ Nesse período houve grande investimento em novas tecnologias pela indústria, principalmente no aperfeiçoamento das imagens como os sistemas de mapeamento eletroanatômico, desenvolvimento de novos cateteres e novas energias para produzir lesões mais efetivas e duradouras e também com o uso do ecocardiograma intracardíaco (EIC) para adicionar segurança na realização do procedimento ao permitir de maneira instantânea a visualização das estruturas do coração e suas relações com os cateteres e bainhas.^2 , 3^

O EIC é uma técnica de imagem invasiva que utiliza um cateter especializado em fornecer as imagens ultrassonográficas a partir de uma sonda que está localizada dentro do coração. Apresenta excelente definição de imagem capaz de reconhecer com detalhes o local da fossa oval, localização dos óstios das veias pulmonares. Permite acompanhar o posicionamento e contato adequados da ponta do cateter com as estruturas de interesse durante a aplicação de RF, assim como a monitorização da formação de trombos ou microbolhas e alterações na refringência tecidual que precedem a formação dos chamados “Steam-pops”.^4^ Outra vantagem do EIC é que é operado em campo pelo próprio eletrofisiologista, sem a necessidade do ecocardiografista.

A aplicação mais importante do eco intracardíaco na ablação de fibrilação atrial está em guiar a punção transeptal com segurança,^5^ inclusive em pacientes sob anticoagulação com INR terapêutico.^6^ Entretanto os benefícios não são restritos à punção transeptal; o EIC permite reconhecer a relação do esôfago com o átrio esquerdo com os locais de aplicação de RF na parede posterior, guiar a posição dos dispositivos de monitorização de temperatura esofágica reduzindo a chance de ocorrência de lesões esofágicas e identificar a formação de trombos intracavitários durante o procedimento.^7^

Uma tendência recente nos laboratórios de eletrofisiologia é reduzir ao máximo a exposição ao RX do paciente e da equipe médica e auxiliares. O uso do mapeamento eletroanatômico foi fundamental nesse processo e atualmente com a associação do EIC, é possível a realização do procedimento sem fluoroscopia. Além dos benefícios da redução da exposição ao RX o método “zero fluoro” permite reduzir o risco de problemas ortopédicos pela não necessidade dos aventais de chumbo.^8 , 9^ Além destas, uma aplicação validada recentemente é a utilização do EIC para a investigação da presença de trombos, principalmente no apêndice atrial esquerdo, antes da realização de ablação de FA, prescindindo do eco tranesofágico. A técnica foi validada recentemente com o posicionamento do cateter do EIC na artéria pulmonar com demonstração de equivalência quando comparada com o eco transesofágico.^10 , 11^

Nos Arquivos Brasileiros de Cardiologia, Sant`Anna et al.,^12^ demonstraram em um estudo clínico observacional, não randomizado, comparando 13 pacientes onde foi utilizado o EIC com 36 pacientes em que foi utilizado o eco transesofágico durante o procedimento. O principal achado foi que o tempo de procedimento foi menor quando o EIC foi utilizado (129±27 min e 189±41min; p<0,001), com menor dose de radiação (51296±24790mGY e 75874±24293; p=0,002). A principal limitação desse estudo é que não houve randomização na inclusão dos pacientes, sendo a seleção baseada na liberação ou não do EIC pelo seguro saúde; contudo os autores demonstraram que as características basais dos pacientes não foram diferentes entre os grupos. Nessa mesma série não foi observado maior risco de complicações ou mudanças no tempo de internação hospitalar, também não sendo possível avaliar nessa série a taxa de recorrência após o procedimento. Apesar dessas limitações os resultados são coerentes e confirmados por outros estudos clínicos^13^ e metanálises.^14 , 15^ Vale destacar que a maioria desses estudos também apresentam a mesma limitação da não randomização e os melhores resultados podem refletir uma melhor curva de aprendizado.

Uma importante limitação para a implementação do EIC nos procedimentos eletrofisiológicos tem sido o custo adicional da sonda do ecocardiograma, que é utilizada em grande parte dos serviços apenas uma vez, seguida do descarte da mesma. Essa limitação pode ser atenuada pelo seu reprocessamento, uma vez que utilizando protocolos adequados, a sua reutilização é permitida no Brasil.^16^ Outro ponto importante é o fato de que no nosso país ainda não faz parte do ROL dos materiais de liberação obrigatória na ablação de FA, portanto algumas operadoras de saúde ainda não liberam o procedimento apesar das evidências demonstrarem o benefício, inclusive sendo esse o motivo nessa série da separação dos pacientes em dois grupos, sendo eles selecionados para uma ou outra tecnologia quando a operadora liberava ou não os cateteres.

Todo avanço tecnológico é bem-vindo e sua implementação deve considerada, principalmente quando pode tornar os procedimentos mais seguros e eficazes. Entretanto, essa decisão deve levar em conta a viabilidade institucional baseada na relação custo-efetividade e o impacto econômico da sua aplicação. Essa informação ainda é necessária para a utilização mais ampla do EIC nos procedimentos de ablação de FA que deve ser compartilhada com as operadoras de saúde e os órgãos responsáveis do Ministério da Saúde que arcam com os custos desses procedimentos. Nesse sentido, estudos clínicos com avaliação custo-efetividade realizados aqui no Brasil ainda são necessários para dar suporte para a sua utilização justificada, assim como a de outros procedimentos de alto custo.
