# Supplementary material for: O Ecocardiograma Intracardíaco Deve Ser Utilizado em Todo Paciente Submetido a Ablação de FA?
Source: Arq Bras Cardiol. 2023 Jun 2;120(5):e20230290. [Article in Portuguese] doi: 10.36660/abc.20230290 (PMC10263402; doi:10.36660/abc.20230290)
Supplement: Translation

# Translation: Intracardiac Echocardiography Must Be Used in All Patients Who Underwent AF Ablation?

**Authors:** Cristiano F. Pisani, Mauricio Scanavacca

**Affiliations:** 1 Brasil Unidade Clínica de Arritmia do Instituto do Coração (InCor) do Hospital das Clínicas da FM USP (HC-FMUSP), São Paulo, SP – Brasil

**Keywords:** Arrhythmia Cardiac, Atrial Fibrillation, Echocardiography/methods, Catheter Ablation/methods, Echocardiography Transesophageal/methods, Ablation Techniques/trends

Atrial fibrillation ablation has become a widely used technique in treating patients with atrial fibrillation in the last 20 years due to better knowledge of its pathophysiological mechanisms, evolution in techniques for isolating pulmonary veins, and reduction of complications associated with the procedure.^1^ During this period, there have been large investments in new technologies by the industry, mainly in the improvement of images such as electroanatomical mapping systems, development of new catheters and new energies to produce more effective and lasting lesions and also with the use of intracardiac echocardiography (ICE) to add safety in the performance of the procedure to the allow instant visualization of heart structures and their relationships with catheters and sheaths.^2 , 3^

The ICE is an invasive imaging technique that uses a specialized catheter to deliver ultrasound images from a probe that is located inside the heart. It presents an excellent image definition capable of recognizing in detail the location of the oval fossa and the ostia of the pulmonary veins. Allows monitoring the proper positioning and contact of the catheter tip with the structures of interest during RF application, as well as monitoring the formation of thrombi or microbubbles and alterations in tissue refraction that precede the formation of the so-called “Steam-pops”.^4^ Another advantage of the ICE is that it is operated in the field by the electrophysiologist himself, without the need for an echocardiographer.

The most important application of intracardiac echo in atrial fibrillation ablation is to safely guide the transseptal puncture,^5^ even in patients under anticoagulation with therapeutic INR.^6^ However, the benefits are not restricted to transseptal puncture; ICE allows recognizing the relationship of the esophagus with the left atrium with the RF application sites on the posterior wall, guiding the position of esophageal temperature monitoring devices, reducing the chance of occurrence of esophageal injuries and identifying the formation of intracavitary thrombi during the procedure.^7^

A recent trend in electrophysiology laboratories is to reduce the exposure to X-rays of the patient and the medical staff and assistants as much as possible. The use of electroanatomical mapping was fundamental in this process, and currently, with the association of ICE, it is possible to perform the procedure without fluoroscopy. In addition to the benefits of reducing exposure to X-rays, the “zero fluoro” method allows for reducing the risk of orthopedic problems by not requiring lead aprons.^8 , 9^ In addition to these, a recently validated application is the use of ICE to investigate the presence of thrombi, mainly in the left atrial appendage, before performing AF ablation, dispensing with transesophageal echo. The technique was recently validated with the positioning of the ICE catheter in the pulmonary artery, demonstrating equivalence when compared with the transesophageal echo.^10 , 11^

In the *Arquivos Brasileiros de Cardiologia* , Sant`Anna et al.^12^ demonstrated in an observational, non-randomized clinical study, comparing 13 patients in which ICE was used with 36 patients in which transesophageal echo was used during the procedure. The main finding was that the procedure time was shorter when ICE was used (129±27 min and 189±41min; p<0.001), with lower radiation dose (51296±24790mGY and 75874±24293; p=0.002). The main limitation of this study is that there was no randomization in the inclusion of patients; the selection is based on the release or not of the ICE by the health insurance; however, the authors demonstrated that the baseline characteristics of the patients were not different between the groups. In the same series, no greater risk of complications or changes in the length of hospital stay was observed, and it was also not possible to assess the recurrence rate after the procedure in this series. Despite these limitations, the results are consistent and confirmed by other clinical studies^13^ and meta-analyses.^14 , 15^ It is worth noting that most of these studies also have the same limitation of non-randomization, and better results may reflect a better learning curve.

An important limitation of implementing ICE in electrophysiological procedures has been the additional cost of the echocardiogram probe, which is used in most services only once, followed by its disposal. This limitation can be mitigated by its reprocessing since using appropriate protocols allows its reuse in Brazil.^16^ Another important point is the fact that in our country, it is not yet part of the ROL of materials for mandatory release in the ablation of AF, so some health operators still do not release the procedure despite the evidence demonstrating the benefit, including this being the reason in this series for separating patients into two groups, being selected for one or another technology when the operator released or not the catheters.

Every technological advance is welcome, and its implementation should be considered, especially when it can make procedures safer and more effective. However, this decision must consider the institutional viability based on the cost-effectiveness ratio and the economic impact of its application. This information is still necessary for the broader use of ICE in AF ablation procedures, which must be shared with healthcare providers and the responsible bodies of the Ministry of Health that bear the costs of these procedures. In this sense, clinical studies with cost-effectiveness evaluation carried out here in Brazil are still necessary to support its justified use and that of other high-cost procedures.
